# The Break-Up Check: Exploring Romantic Love through Relationship Terminations

**DOI:** 10.1007/s11406-017-9935-8

**Published:** 2017-12-22

**Authors:** Pilar Lopez-Cantero

**Affiliations:** School of Social Sciences – Department of Philosophy, The University of Manchester, Arthur Lewis Building, 4th floor, M13 9PL, Manchester, UK

**Keywords:** Emotion, Romantic love, Break-ups, Relationships, Value, Grief

## Abstract

People who experience love often experience break-ups as well. However, philosophers of love have paid little attention to the phenomenon. Here, I address that gap by looking at the grieving process which follows unchosen relationship terminations. I ask which one is the loss that, if it were to be recovered, would stop grief or make it unwarranted. Is it the beloved, the reciprocation of love, the relationship, or all of it? By answering this question I not only provide with an insight on the nature of break-ups, but also make a specific claim about the nature of love. I argue that the object that is universally lost in all break-ups is a person with certain intrinsic qualities, who is in a relationship characterised by certain shared activities and recognized as romantic. That means that, at least in romantic terminations, the beloved and the relationship are not independent objects of grief. So, plausibly, they may not independent objects of value in love. Hence, those who state otherwise (within the property view and the relationship view) should face up to this objection coming from the study of break-ups.

In her study of romantic love, Brogaard describes the phenomenology of romantic break-ups:

“After being dumped by the one you thought you would grow old with, you may feel your guts being sluggishly cut out of you with a butter knife and squeezed through a meat grinder, hear your beau and his voices in your head, and feel your sand papery cheeks dissolving in tears” (2015:72).

Indeed, many people are familiar with the emotional pain that sometimes follows a relationship termination. Break-ups can hurt for many different reasons. In this paper, I look into the nature of break-ups in order to find a loss which is grieved in all romantic terminations.

I start with a generic definition of love and a summary of the property view and the relationship view which I will scrutinise through the lens of break-ups. Then, I offer a taxonomy of terminations and give a brief account on unchosen break-ups as grieving processes, before moving on to my main question. Specifically, I ask what would be the object that would have to return after an unchosen break-up in order to stop or unwarrant the grieving process.

Firstly, I consider the object that is valued in love according to the property view: a particular person with certain intrinsic qualities. Secondly, I consider reciprocity. Thirdly, I consider the relationship understood as shared activities performed out of concern, which is the object of value in the relationship view of love.

Going through each potential loss, I show that the return of the person would not stop or unwarrant the grief if she does not return to a relationship where reciprocated love is expressed in certain activities, and which is romantic in virtue of certain expectations. That is the universal loss in all break-ups.

In the last section, I argue that since the person and the relationship are not independent objects of grief, plausibly they are not independent objects of value either. That means that the property view and the relationship view of love should not stand in opposition to each other, at least not without facing up to this objection coming from the study of break-ups.

## Love and the End of Relationships

In order to ask specific questions about terminations, I shall start by giving a generic definition of love. Despite disagreement on the nature of love, there are two elements which a large number of views take into account: concern and value.^1^ Going into the multitude of views here would sidetrack my argument, so for the purposes of this paper my generic definition will be the following:

Loving is a kind of valuing another person that involves or consists in concern for the beloved. That concern is (1) non-instrumental and (2) personal.

I will not be discussing whether concern is a constituent of love, as portrayed in the *love-as-valuing views* (cf. Kolodny 2003) or analogous to what love is, as depicted in the *love-as-concern views* (cf. Frankfurt 1999; Soble 1990). My generic definition is not in opposition with either –I say love “involves or consist in” concern.

Regarding non-instrumental concern, I will assume that loving someone non-instrumentally is compatible with the expectation of reciprocity. The need for reciprocity has led some philosophers to regard romantic love as an impure type of love (Frankfurt 1999:43; Velleman 1999:352.). If loving someone romantically is grounded or conditional on a desire for having that love reciprocated, the argument goes, that someone is not loved in a non-instrumental or disinterested way. However, that argument does not take into account the difference between romantic love and romantic relationship. Protasi explains that reciprocity is a necessary element of romantic relationships: if love is not reciprocated, it is impossible to enter a loving relationship as a romantic partner in the first place (2014:217). It would be possible to have a relationship without reciprocity, but it would not be a loving relationship. Protasi calls this type of partnerships “social relationships”: “socially regulated, or even institutionalized, practice[s], such as dating, marriage, and the like” (2014:215). These can exist without love, but are easily confused with loving relationships because people who love each other often engage in these practices. Nevertheless, the requirement for reciprocity does not entail that people in romantic relationships are loved instrumentally, since the lover’s appreciation of the beloved is *not conditional on* being reciprocated (Protasi 2014:218; italics mine). That is, love for the beloved does not disappear if it is not reciprocated. Then, reciprocity is necessary for romantic relationships, but not for romantic love. That explains how people can experience unrequited love.

There are a few different interpretations of what it means for concern to be personal. Here I will deal with two positions within the love-as-valuing perspective: the property view and the relationship view. The *property view* is the view that ‘loving someone for who they are’ is loving them for the intrinsic qualities that make them who they are. For example, that they are charming and loyal –as opposed to other qualities which are not intrinsic, like say that they eat a huge amount of pizza and they have blond hair. According to the *relationship view*, ‘loving someone for who they are’ is loving them for the relationship we have with them, consisting in shared activities.^2^ For example, that they are our mother, our sister or our romantic partner.

The property view and the relationship view are both aimed at finding the justification of love. That is, they are focused on finding the *reasons* which ground love. They are mutually exclusive: according to the property view, it is the intrinsic qualities of the beloved which grounds love. According the relationship view, it is the relationship with them which grounds love. In the property view, the relationship is still an object of value, but it is not the ‘central element’ that justifies love. Equally, in the relationship view, the beloved’s intrinsic qualities are still valued in virtue of the beloved herself being an object of value, but they are not the central element that justifies love.

The justification of love is not the focus of this paper. Neither is the discernment of the ‘central element’ in love. In other words, I will not ask what single element makes love, or break-ups, appropriate or *rational*.^3^ Instead, I will ask what makes break-ups *painful*. I will look at the nature of relationship terminations in order to find out if the beloved –understood as a person with certain intrinsic qualities– and the relationship –understood as shared activities– are two objects which are grieved in all break-ups. I will also ask whether they are grieved independently, as we would infer from their apparent mutual exclusivity as objects of value. Before I give more details on how I will proceed, I turn to my description of romantic terminations.^4^

A termination entails the end of a relationship. But relationships, as break-ups, appear to be very heterogeneous. I categorise romantic terminations into the following two types:

i) Death. When one party dies, the relationship is terminated against the other party’s desire to continue the relationship in virtue of her desire of the deceased to continue living.
ii) Break-up. When a relationship ends for a cause other than death, there are two possible ways of experiencing the termination:

- Unchosen. The party does not want to end the relationship, but the other party makes that decision independently of her desire to continue the relationship. This type of break-ups can be experienced only by one member of the couple.
- Chosen. The party wants to end the relationship. This type of break-up can be experienced by one or both members of the couple at the same time, in two further distinct ways:
  - Chosen break-ups are *pursued* when the party who ends the relationship acts on the desire of not wanting to be in the relationship anymore.
  - Chosen break-ups are *accepted* when the party who ends the relationship has the desire to continue the relationship, but acts on a different desire (i.e. long-term well-being).^5^

I will only focus on unchosen break-ups. The emotional pain which usually follows unchosen break-ups can be isolated in a way that is not possible in other types of terminations. Chosen break-ups can be painful, but describing the emotional pain in these cases would be impossible without dealing with the rationality of love, the rationality of desire and the rationality of emotional pain in themselves. Since, as I have said, I am leaving rationality to a side, I will not discuss chosen break-ups.

Finally, it may seem that whatever I say about unchosen break-ups may apply to deaths too. Unchosen break-ups entail the loss of certain features of one’s life that one did not renounce by choice. Deaths do too. But while death implies an irreversible loss of the beloved, it is not clear that break-ups do. I deal with the specific question of the loss of the beloved in section 3. Instead of looking at terminations by assuming that they entail the loss of a person, I take a step back and ask whether they do entail that at all.

All things considered, from now on I refer only to unchosen break-ups unless explicitly stated otherwise. My aim is to find out what is grieved in break-ups, so next I offer support to the claim that break-ups can be a source of emotional pain.

## Break-Ups and Emotional Pain

When someone is in a relationship that she values with a person that she loves, the termination of the relationship entails the loss of many things that she values. Some of those things are valued instrumentally, that is, for the sake of something else. For example, the loss of financial security, the loss of someone who proof-read her philosophy papers or the loss of the only person who would accede to go to comic-cons dressed up as Chewbacca when she went as Han Solo. Some other losses have intrinsic value, that is, are valued by themselves beyond any utility they may have. For example, after being abandoned some people will suffer due to the loss of self-esteem (Ben Ze’ev and Goussinsky 2008:41) or validation (Milligan 2011:45).^6^

When something of value is lost, that loss often triggers a characteristic response of emotional pain: grief.^7^ Not all losses of valued objects warrant a grieving response. “In grief, one sees an *important* object or person as lost” (Nussbaum 2001:28, italics mine). The objects which may be important enough to ground a grieving response are varied and personal: it can be “the loss of a job, a limb, a breast, a home, a relationship” (Brogaard 2015:24). I will leave that concept of importance open and assume it can be subjectively justified. Two people can value an object equally (say, for example, their shared car) and the loss of it may trigger grief only for one of them (if that car was important for her).

For Nussbaum, an episode of grief combines the judgement that an object of value has been lost with a wider network of values about the world one inhabits (which is now changed by the loss) (2001:76). Goldie describes grief from a different outlook. He argues that while emotions like fear might be episodic (one has fear in the moment one is afraid), grief is an emotion which unfolds over time in the shape of a process. That means that someone is still immersed in the grieving process even when she is not experiencing emotional pain at every single moment of such process (2012:61).

The process of emotional pain after an unchosen break-up fits well with Goldie’s definition of a grieving process. After a termination, a person may not feel grief, for example, when she is trying to pick a cereal brand in the supermarket. But that does not mean she is not in a grieving process. She may still feel grief when she walks by the bar where she had her first date with her beloved, or she realizes it is her former partner’s birthday. It also seems that the episodes that make up this process are the kind of episodes described by Nussbaum: we not only evaluate the loss of our object of value as unfortunate, but it seems like everything that we look at is somehow affected by that loss. It is then plausible that break-ups are grieving processes in the sense described. Giving a comprehensive list of the losses caused by break-ups would be impossible, because they will be particular to each relationship. What I will do here is try to find losses which can be universalizable to all break-ups, without claiming that those universalizable losses are the only things that may be lost.^8^

Before developing my answer, I shall respond to an objection: not all unchosen break-ups hurt. Such claim would be in contradiction with the fact that the emotional pain triggered by relationship terminations is widely supported by empirical studies.^9^ I can provide with additional answers stemming from the nature of grief.

Firstly, grief sometimes goes unnoticed as the result of an epistemic failure. For example, holding the false belief “I am not grieving” when one actually is. In a related example, someone may falsely believe that they loved their partner and they wanted to continue the relationship, when they actually did not do either. The termination, although not initiated by them, would not go against their desires: it would be a chosen break-up, which can also be painful –but those are not the matter of my discussion.

Secondly, people who at first glance do not seem to be grieving may just be having a non-paradigmatic response to grief. We tend to think that a person is only grieving if they look grim and report or express an intense feeling of sadness (for example, if we see them crying). But there are responses to emotions which do not fall in the common paradigms (Goldie 2012:62). After a break-up, some take up on a dating spree, others concentrate their efforts on chasing the person who left them, and others throw themselves into translating Hume’s works into Sumerian. These may all well be grieving responses and expressions of emotional pain, albeit non-paradigmatic.

All of the above does not prove that all unchosen break-ups hurt. But it offers a plausible answer to why sometimes they do even when it does not seem so. I will, however, refrain from claiming that all break-ups hurt. There may be cases when they do not. However, I can still ask why break-ups hurt when they do.^10^

I will proceed in the following way. If the loss of a valued object is what grounds grief, it is plausible to infer that the return of said lost object will stop the grief, in virtue of having erased the loss. At least, that is true in cases of death. There is a good example of this situation in BBC's *Sherlock* (spoilers follow). At the end of season 2, Sherlock dies after falling from a building. That leaves Watson devastated. He grieves the death of his dear friend for a long time, and is seen visiting Sherlock’s tombstone. However, at the beginning of season 3 we are shown that Sherlock faked his death in order to get rid of Moriarty. When Sherlock shows up again and reveals the truth, Watson is not grieving anymore. Sure, he is immensely angry with Sherlock for having done that to him, and their relationship is stranded for a while because of it –actually it has a lasting effect on his attitude towards Sherlock even after they have reconciled. But he is not grieving anymore –at least not over the same object.

From this example, we can infer that the grieving over the lost object stops when the lost object is returned. Watson could continue grieving, say, the loss of Sherlock’s loyalty (since he has lied to him). But that grief would be independent of his grieving the loss of his friend. If Watson continued grieving the loss of Sherlock, his grieving would not be more warranted than him grieving the loss of his daughter (who is alive).

In the following sections, I will ask whether the same happens in break-ups. I will work under the two principles I have laid out in this section. Firstly, all break-ups entail the loss of a valuable object which is the object of grief. I will call that object X. Secondly, the return of object X stops or unwarrants the grief over the loss of it.

I will start from an intuitive answer to the question of why are break-ups painful: they entail the loss of a partner who we love. Specifically, a person who we love, and who was in a loving relationship with us, does not want to continue the relationship. Here we have the two concepts (person and relationship) which the opposing property view and relationship view consider independent objects of value which ground love. There is also a third concept: reciprocity. What I will do is look at those three possible objects of grief to determine whether they are universal to all break-ups, whether their return would stop or unwarrant the grief; and whether these losses are valued independently.

Hence, in the next three sections I will ask whether any of the following statements is true:

(A) X is a person with certain intrinsic qualities.
(B) X is reciprocity.
(C) X is a relationship constituted by shared activities.

## Grieving the Beloved

In everyday language and in artistic representations of love, we do talk about break-ups as if they entailed the loss of a person. Take, for example, Heathcliff’s exclamation to Catherine in *Wuthering Heights*:

“Be with me always—take any form—drive me mad! Only do not leave me in this abyss, where I cannot find you! Oh, God! It is unutterable!”

Indeed, people who go through break-ups usually say missing their beloved is a source of emotional pain. It seems then that (A) is intuitively true. However, (A) is problematic in different ways.

There is a trivial objection to the claim that the beloved is lost after a break-up that we can safely ignore: they are still alive. When someone breaks up with their partner they do not die. They can always return –like Sherlock. However, we can ignore this response because the grieving party may experience a loss which appears indistinguishable to the death of the beloved; for instance, when all contact is severed between ex-partners after a termination. Though the beloved is not dead –they continue existing– they do not exist for the grieving party. It seems unprincipled to deny that some people experience break-ups in this way. Whether or not the person is actually dead appears to have little bearing on the emotional pain experienced. So, I will ignore this potential response. Perceived losses count just as much as actual ones.

A more reasonable response to the claim that (A) is not true is that not all people feel like their former partner has died after the break-up. Indeed, many people continue to keep in touch with each other after a termination. By this, I do not mean the phone calls between a pair of divorcees regarding the dog’s custody, but more meaningful contact. Some ex-partners remain close. Some keep celebrating holidays, birthdays and anniversaries. There are even cases of people who get divorced and continue living in the same house. In all these cases, it does not seem that the beloved has been lost and can thus be the object of grief.

Hence, it seems that (A) may not be true because the beloved does not seem to be an object of grief which is universal to all break-ups. I would argue, however, that this is the wrong conclusion to make from cases where ex-partners continue in contact. I will provide my answer to this objection, which also presents a challenge for (B) and (C), further on in the paper. In this current section, I will respond to a different objection to (A).

Let us look at the following situation, where T1 is the moment of the break-up and T2 the moment where the beloved returns:

[T1] Aurora and Diana were in a romantic relationship until Aurora broke up with Diana. After the break-up, Aurora disappears. Diana did not want to break up and still loves Aurora. She loves her for who she is: a charming woman who is above all loyal. She experiences intense emotional pain because of the break-up and she misses Aurora everyday. For her, it feels like Aurora has died.

[T2] After two months, Diana is still suffering and loving Aurora like the first day. But one morning, Aurora shows up at Diana’s door: “I want to be in your life again. I miss you. Please let me come back home”. “Does this mean that you want to be together again?”, asks Diana. “No”, says Aurora. “I only love you as a friend. I do not want to be your partner; just to be in your life”.

At T1, Diana is experiencing the perceived loss of an object of value: Aurora, who she values in virtue of her intrinsic properties of being charming and loyal. It seems like the object of grief is the beloved –(A) seems true at T1.

Moving to T2, the loss of the apparent object of grief is restored (as in the examples where ex-partners continue having contact with each other). Aurora is back: “I want to be in your life again. I miss you. Please let me come back home”. She is as charming and loyal as always. Imagine that she allowed Aurora to move back in. Would she still be grieving even when her beloved is next to her everyday? I believe she would. In fact, Aurora coming back and not wanting to re-start the romantic relationship, with Diana having to live with her everyday knowing that she only loves her as a friend, may even intensify Diana’s emotional pain. So (A) does not seem true not only because the beloved has not been lost, but because her return does not stop or unwarrant the grief.

The cases above, however, only seem to disprove (A) if we interpret (A) from the property view (i.e. the loss of a person who is valued in virtue of certain intrinsic qualities). Kolodny has an example where a prestigious historian changes his personality (he becomes a Holocaust denier in order to satisfy his vanity). He makes the following observation about his wife’s plausible reaction:

“Perhaps the person with whom one once had a relationship was a fiction that one confused with this other person. Perhaps the person once existed, but has come to be replaced by this other person. As people say in movies, we might expect the historian’s wife to say: “You’re not the man I married” or “I don’t know who you are anymore”” (2003:165).

The example suggests that someone can change their intrinsic qualities so fundamentally that they can appear to be different people. Going back to my example, imagine that Aurora told Diana that she had been unfaithful. In that case Diana, who knew that fidelity and monogamy were two essential principles for Aurora in virtue of her loyalty, may form the belief that she has lost her beloved (because Diana is not the woman she used to be).

As Kolodny puts it, this is a “psychologically real, but metaphysically vexed, phenomenon” (2003:165). It is metaphysically vexed because it involves a question on personal identity which is highly controversial: can people have changed so much at T2 that they become numerically different to the person they were at T1? There is no need to get into the metaphysical debate on personal identity here.^11^ As I have said, perceived losses count as real losses. So regardless of the metaphysical status of Aurora at T2, I accept that Diana would have felt she had lost her beloved if she does not see her as the same person anymore. In this case, there would not be a return of the actual object of grief, but of a different object.

But then again, why does Diana still grieve if Aurora T1 and Aurora T2 have the same intrinsic qualities? I said that it would be wrong to assume that the answer is that (A) is untrue because the beloved is not universally grieved and her return would not stop the grief. My answer is that even if Aurora is still charming and loyal, the real object of grief has not really returned –just as it did not return in the case of the historian and in the variation of my example where Aurora has been unfaithful. Despite having the same intrinsic qualities, Aurora T2 is not the same person as Aurora T1 because she lacks two features: “I only love you as a friend… I do not want to be your partner”. She lacks her reciprocation of Diana’s love (B) and her romantic relationship with her (C). In other words, (A) is not true independently of (B) and (C). The beloved is not, then, an independent object of grief. But that does not mean that it is not an object of grief, as I show once I give my arguments for (B) and (C).

## Grieving Reciprocity

There are two possible interpretations of what reciprocity means. One way is to understand reciprocity as the return of something that is given. A second way is to understand reciprocity as the mutual contribution to something that is shared. Translated into the issue of love, Krebs has characterised those as the interpretation of reciprocity as “A loves B” and the interpretation of reciprocity of “two people love each other”, respectively (2014:23). Love-as-valuing views use the first interpretation, so I will do the same here.^12^

Intuitively, it seems that the loss of reciprocity (i.e. the fact that our beloved does not love us anymore) can be a source of emotional pain. This is from the song ‘For No One’ by The Beatles:

“Your day breaks, your mind aches/ You find that all the words of kindness linger on/ When she no longer needs you … And in her eyes you see nothing/ No sign of love behind her tears/ Cried for no one/ A love that should have lasted years”.

I said earlier that reciprocity is always a feature of romantic relationships. When two people have been in a romantic relationship and one terminates the relationship because she does not love the other anymore, this other person has experienced a loss of reciprocity. Usually, it is important for people that their partners reciprocate their love, since they want to be in a loving relationship with them. If reciprocity is lost, then, it can be an object of grief. But is it the universal object of grief which would stop the grief if returned? In other words, is (B) true?

First of all, (B) is not true independently of (A). Diana will not stop grieving if reciprocity is returned to her from anyone who is not Aurora T1 (not even by Aurora T2). Reciprocity would not be, then, an independent object of grief. So if (B) is true, it is necessarily concurrent with (A): X would be (B) reciprocity from (A) a particular person with certain intrinsic qualities.^13^

It seems, however, that (B) may not be true, because not all break-ups result in a loss of reciprocity in the way described –some people continue loving their ex-partners despite breaking up with them. In my original example, it is disputable whether Diana has really lost Aurora’s reciprocity. After all, Aurora says that she loves her as a friend. That mirrors many break-ups in the real world. Imagine that Diana accepted Aurora’s suggestion and actually went on to enjoy a meaningful –and reciprocated– friendship with her. This objection is tied to the one I left unanswered in section 3. Does Diana –or people who continue having a meaningful relationship with their ex-partners– experience a loss of reciprocity in that case?

The answer is yes, because Aurora’s love for Diana has changed. If we understand reciprocity as “A loves B”, the love that she is directing at Diana at T2 is not the same that she was directing at Diana when she was in the relationship, and it is not the same as the love Diana has for Aurora at both T1 and T2. Diana has lost the love that Aurora used to reciprocate her with. In everyday language, we would probably say that Aurora’s love for Diana at T2 is not romantic. In section 5, I say more about what ‘romantic’ may mean here –for now it suffices to say that a change in the nature of the love entails a loss of reciprocity.

The above, however, only proves that romantic reciprocity is lost also when people continue loving each other as friends, not that (B) is true. In my example, Aurora tells Diana that she wants to move back in with her: “I want to be in your life”. If Aurora returned having her same intrinsic qualities and reciprocating Diana’s romantic love, but said that she was going to disappear again, would Diana still grieve? If she would, (B) is true.

There is, again, a trivial objection to (B) from this stance. It may be that Diana does not grieve at all, and really goes from being Aurora’s romantic partner to being a friend without any emotional pain. In that case, the loss of romantic reciprocity was not *important* to her. But only important losses ground grief. So if someone substitutes a romantic friendship for a meaningful friendship without any grieving transition, it does not necessarily mean that they did not love them romantically –it may be that romantic reciprocity was not important for them. This would be a case of a break-up that does not hurt, and is thus trivial because it is irrelevant to my discussion on break-ups which are painful.

There is a stronger objection to (B) coming from chosen break-ups. I presented chosen and accepted break-ups as scenarios where the person ending the relationship wants to continue the relationship, but that desire is trumped by another desire. The trumping desire need not be self-regarding, i.e. pursuing one’s own career. It could actually be grounded on love for the other person.

Imagine that Aurora knew that being in a relationship with Diana is preventing Diana from pursuing a career in Hollywood. It could be that Aurora loves Diana intensely, and really wants to be in a relationship with her, but that desire is trumped by the desire for Diana’s long-term well-being (which includes pursuing a career in Hollywood). Imagine that Diana does not agree with this, and does not want to end the relationship regardless of her Hollywood aspirations: she would be experiencing an unchosen break-up. But she would not have lost Aurora’s romantic reciprocation, so it seems that (B) is not true.

However, Diana would still grieve the loss of reciprocity in the above scenario. The answer lies in the nature of reciprocity. Reciprocity is not an independent entity. As I said above, reciprocity is dependent from a particular person. Besides, reciprocity can only be experienced in action. Saying “I love you”, taking care of your beloved when they are ill, or doing a long train trip together every year, are examples of actions which can express reciprocity. Each romantic partner expects different actions from their beloved as the expression of their love (Protasi 2014:216). However, in virtue of being romantic *partners*, one of these expectations is that these actions are performed within a *romantic relationship*. Diana and Aurora are not in such relationship anymore, so for Diana, Aurora’s reciprocated love has changed in virtue of it not being expressed in action within a romantic relationship. Hence, Diana would still grieve if Aurora keeps her intrinsic qualities and still reciprocates her love: (B) is true.

I have shown that the objections to (B) are not valid. Even in cases when love is somehow reciprocated, the grieving party experiences a loss of reciprocity. Since so far (A) and (B) are true, the object of grief X is plausibly a person with intrinsic qualities and reciprocity from that particular person. But as I have said, Diana would not stop grieving if Aurora returned having the same intrinsic qualities, but she did not express her love in certain actions. In other words, she would not stop grieving if the relationship does not return too.

## Grieving the Relationship

Kolodny says that a loving relationship is an ongoing pattern of concern for a particular person, which grounds the performing of shared activities which are characteristic of the relationship (2003:147). It seems intuitive to think that the loss of shared activities can be a cause of emotional pain after a break-up. In this section, I argue that (C) is true: the relationship is an object of grief which is universal to all break-ups, and which would stop the grieving if returned. But (C) is inseparable from (A) and (B). Diana would not stop grieving the loss of Aurora if she entered the relationship with someone else. Equally, as seen above Diana would not stop grieving if she entered a relationship with Aurora where she does not reciprocate her love. Hence, I will consider if the object of grief X is a combination of (A), (B), and (C); not (C) alone.

According to Kolodny, when we talk about ending a relationship we often take that to involve “deciding to stop engaging in [shared] activities: deciding to move out or no longer see each other socially” (2003:149). But in the previous sections, I have presented a potential objection to (C): people may break-up and still engage in the same shared activities afterwards. If Diana and Aurora move back in together and continue going to their yearly train trip, have they lost their relationship? I believe they have. Even if they do the exact same things that they used to do before the break-up, there is a loss of reciprocity, which means that the relationship they have now is a new relationship. That means that the relationship is a universal loss in break-ups, since it is lost even in situations where people do not stop engaging in shared activities and keep reciprocating concern. In other words, that objection to (C) is not valid.

My observation above hinges on a claim that I made in section 4: the change of the nature of love entails the loss of romantic reciprocity, which in turn resulted in the change of the nature of the romantic relationship. I said that after a break up, love is not ‘romantic’ anymore. That probably seemed an intuitive claim, since traditionally friendship and romantic love are considered to have different nature by being different types of love. Kolodny himself takes the approach of differentiating romantic love from friendship as different “modes” which are expressed through certain characteristic activities (2013:147). However, I have claimed that activities are ‘romantic’ as long as they are performed within a romantic relationship. That departs from the intuitive view of modes of love and poses a threat of circularity. So, in order to show that (C) is true, I need show that my claim on ‘romantic’ action is correct.

Kolodny’s claim of ‘romantic’ being just a particular ‘mode’ of love faces the challenge of one of the examples I set above. I have shown that if the object returned to Diana is a person with certain intrinsic qualities (Aurora) with whom she does the same things that she used to do (the relationship understood as shared activities), Diana would still grieve. Kolodny cannot explain why Diana would grieve even if she keeps her beloved and the relationship. I can: the nature of her love has changed; not insofar the characteristic activities have changed in content (like Kolodny woud suggest), but insofar the activities have changed nature.

What is the ‘romantic’ nature of actions which are performed within a romantic relationship? It lies on a missing (but plausibly implied) element in Kolodny’s theory: romantic expectations. I have said in the previous section that expectations vary, but some are expressive of love within a romantic relationship. I will give a last example to show that (C) is true if understood as shared activities performed out of concern and *the fulfilment of expectations recognized as romantic.*

Diana had certain expectations from Aurora when they were in a relationship. Some were socially acquired (like being monogamous), some were particular to the relationship (like doing their yearly train trip) and some were particular to Aurora (like her returning home everyday before 9 pm). Those were romantic insofar they are the expression of the expectation of romantic reciprocity *for Diana*. Diana’s expectations were warranted when Aurora is acting on the desire “I want to be your romantic partner”. However, in the example, Aurora says “I do not want to be your partner”. If Diana accepted going into a new relationship with Aurora, she could not say anymore she is in a romantic relationship with her, since Aurora has not accepted that. The only thing that would have changed in this extreme case (where someone breaks-up but continues the relationship exactly as it was, and does not change her personality) is that the relationship would not be recognized as romantic anymore. There would have been a loss of recognition, in virtue of which some expectations will have been lost –so the relationship would have been lost. Diana would still grieve the loss of the relationship. The loss of recognition as romantic would have changed the relationship, so she would have lost the relationship she had. Hence, (C) is true even in cases when everything stays the same after the break-up but the relationship is not recognized as romantic anymore.

## Conclusion

From my arguments above, one possible answer to the question of what is the object which is grieved in all break-ups and which would stop the grief if returned (X) is the following:

X= (C) a recognized romantic relationship characterised by shared activities which are the expression of (B) reciprocated love from (A) a particular person with certain intrinsic qualities.

That would be an interpretation of break-ups from the point of view of the relationship view. However, there is another possible answer which would be the interpretation of the property view:

X: (A) a particular person with certain intrinsic qualities and the relational property of (C) being in a recognized romantic relationship characterised by shared activities which are the expression of (B) reciprocated love.

What these two interpretations have in common is that they reflect that the beloved and the reciprocated relationship are not independent objects of grief. Actually, these two ways of interpreting X are analogous. They merely shift the perspective from the person to the relationship because they stem from views where the aim is to find the central element of love. But once we look at love from the perspective of terminations, it is patent that such approach misrepresents the phenomenon. If grief is warranted by the loss of a valued object, and the objects of value from the relationship view and the property view are not grieved independently, then they are not valued independently either. If love is valuing, valuing a person and valuing a relationship have to be interdependent. There is no ‘central element’ of valuing in the same sense there is no ‘central element’ of grieving. I believe that instead of separating the objects of value, we should strive for an integrating theory of love which accommodates this.

Defenders of the property and the relationship view may have possible answers on how, if the objects of value are not individuated and one of them signalled as the central element, there are certain challenges to be met. They would all be challenges from the perspective of rationality –whether we would still be rational in loving a particular person and not trading-up or changing them for a doppelganger, for instance. They could say an integrated theory would have to concede that it would be rational to change or trade-up, for example. The question is –does it really matter whether it would be rational? Many things we can potentially do are rational, but that does not mean that we want to do them, or that we can do them without a cost that we just do not want to pay (for example, losing your actual partner in order to ‘trade-up’). In any case, my aim was to lay down a challenge for these love-as-valuing views while offering some insights on the nature romantic break-ups. So, indeed, the challenge has been set: it may be time to give up the ‘central element’ approach, unless its proponents have a better account on the grieving process which break-ups amount to.

## Footnotes

1 This is not true of all views. Darwall sees love as a second-personal attitude which takes the form of “addressing” the other (2016:175). Velleman argues that love as a way of expressing respect for her as an autonomous being (1999). Harcourt (2016) and Wonderly (2017) have developed two different views of love as attachment. For the purposes of this paper I classify these, too, as ways of valuing another person. There is, however, a cluster of views where valuing is not seen as a central element of love at all: the so-called identification theories, according to which love is a way of identifying with another (cf. Nozick 1989; Ben-Ze’ev and Goussinsky 2008). This paper deals only with a specific set of love-as-valuing views for two reasons. The first one is to show that they are defective if seen in isolation from each other. The second one is to suggest that the ‘central element’ approach may be a wrong one, so maybe the love-and-valuing and love-as-identification views should get closer in the way I claim the property and the relationship love-as-valuing views should. There are also views which integrate the valuing and the identification elements (see for example Helm 2010; Smith 2011). I intend this paper to indirectly act as support for the idea that the study of love should be re-directed with that integrating tendency, instead of the current focus on the search for a ‘central element’.

2 That is Kolodny’s version of the relationship theory. His is not the only one, though: Krebs has developed the so-called ‘dialogical model of love’ which is also focused on the link between the lovers. I refer back to the dialogical model in section 5.

3 For a discussion on the rationality of stopping loving someone from the relationship view, see Kolodny (2003:164–167). Protasi (2014) discusses the reasons of unrequited love. I engage with both their theories throughout the paper, but I neither endorse nor reject their claims on rationality or appropriateness.

4 Romantic relationships are not the only relationships which can be terminated. Friendships can be ended too, and arguably so can familial relationships. My focus here is on romantic terminations only.

5 This taxonomy may seem to not accommodate idiosyncratic cases. One’s partner might die or break-up just before one was going to end the relationship, so the termination would be desired although not initiated by the party. The death of a partner may be desired, for their sake, if they are terminally ill. However, these terminations fit in the generic taxonomy I offer: they just fit into several categories at a time.

6 For an objection against this tendency to connect love with worthiness, see Jenkins’s rejection of “amatonormativity”: the idea that “romantic love is the normal or ideal condition for a human life, so lives that don’t include it are imperfect or abnormal” (2017:65).

7 I will argue under the assumption, supported by philosophical and psychological evidence, that grief is an appropriate response to loss as long as it does not turn into so-called “complicated grief”: “prolonged and intense grief that is accompanied by complications that derail the progress of grief and is associated with substantial impairment in work, health and social functioning” (Zisook et al. 2010:1099). For a philosophical rejection of grief as an appropriate response to loss, see Wilkinson, 2000.

8 I am grateful to an anonymous referee for inviting me to clarify this point.

9 Psychologists consider rejection a powerful source of emotional pain, since it is a direct attack on an almost universal “need to belong”: human beings need to form attachments and to preserve them (Baumeister and Leary 1995:499). In her anthropological studies on unrequited love, Fisher reports that the intense suffering resulting from a love rejection is similar to the physical and psychological pain of withdrawal syndrome (2014:240). For a wider discussion of empirical research on break-ups and unrequited love from a philosophical standpoint, see Brogaard 2015:12–38. I am grateful to Helen Beebee for pressing on this objection.

10 I will also remain agnostic as to whether grieving is the only appropriate response to loss. Solomon argues that it is indeed morally obligatory to grieve the loss of a lost one as the expression of our love to them (2004:78), thus creating a crucial ethical connection between grief and love (love entails a debt of grief towards the beloved). In these lines, it can be argued that some people who are not grieving actually should be grieving, so they are not assessing the situation properly (since if they did, they would grieve). My claim is independent of the moral obligatoriness of grief.

11 Love-as-identification views could contribute a lot into this question, since according to them loving someone changes who you are (so plausibly, so would do stopping loving someone). I am dealing exclusively with love-as-valuing views here, so I assume that the beloved needs the exact object of value to return in order to stop grieving.

12 Views as Krebs’s and Smith’s, which focus on the link between lovers, offer a different insight into the grieving process. If reciprocity is understood as a pooling of some elements (action and feeling/concern), there is a loss of that pooling itself. Krebs identifies that link as “love proper” (2014:23); for Smith it is some sort of shared object of love in the Nozickian sense (“the two of them, collectively” (2011:79)). In that case, here there are two other possible losses which are universal to all break-ups. It would be an interesting project, but these views are beyond my discussion here insofar they are *already* integrating views (i.e. not looking for a single ‘central element’). As I said, I want to offer a reason for the justification theories to stop focusing on a central element and its bearing on rationality: I am taking a step back, so I cannot bring these views into the current discussion.

13 Surely, often look for a new relationship after a break-up, which seems to contradict the claim that reciprocity cannot come from anyone. That is the so-called ‘fungibility problem’ (Helm 2010:25), according to which the object of one’s love is fungible, interchangeable with someone else with the same qualities. Fungibility is only a problem from the perspective of rationality –it would be rational to love the substituting object. I am not dealing with the rationality of love, but with the loss of the person with certain qualities and her reciprocity. So it is irrelevant for my purposes whether those can be actually substituted for someone else without being irrational. Even if fungibility is really a problem, the object of love cannot be traded *without loss* (I owe that point to Edward Harcourt).
